# Supplementary material for: Modeling confirmation bias and polarization
Source: Sci Rep. 2017 Jan 11;7:40391. doi: 10.1038/srep40391 (PMC5225437; doi:10.1038/srep40391)
Supplement: Supplementary Information [file srep40391-s1.pdf]

Supplementary Information for
Modeling confirmation bias and polarization

Michela Del Vicario, Antonio Scala, Guido Caldarelli,
H. Eugene Stanley, Walter Quattrociocchi

August 24, 2016

S1 Simulation Results for RBCM

The RBCM differs from the standard BCM in the first phase, where a series of random rewiring steps is performed until all links in the network are concordant, meaning that the difference between the opinions of the two endpoints of each link is smaller than ε. Through this procedure we obtain a network in which all randomly chosen pairs of users interact and hence the consensus is reached also for smaller values of ε. In Fig. S1 we show the estimated mean number of steps needed to get the fully concordant network as a function of ε, where the results are averaged over 50 realizations. The decay of the estimated mean number of steps is best fitted by the power law ax^{-b}, where the parameters $a = 5.105$ and $b = 1.072$ are obtained through Nonlinear Least Square (NLS) fitting.

Figure S2 shows the final distribution of peaks for the RBCM for varying $(\varepsilon, \mu) \in [0, 0.5] \times [0, 0.5]$. We notice that consensus is reached for smaller values of ε w.r.t the BCM. Indeed, while for BCM consensus is reached for $\varepsilon \geq 0.25$, for RBCM we get it for $\varepsilon \geq 0.15$.

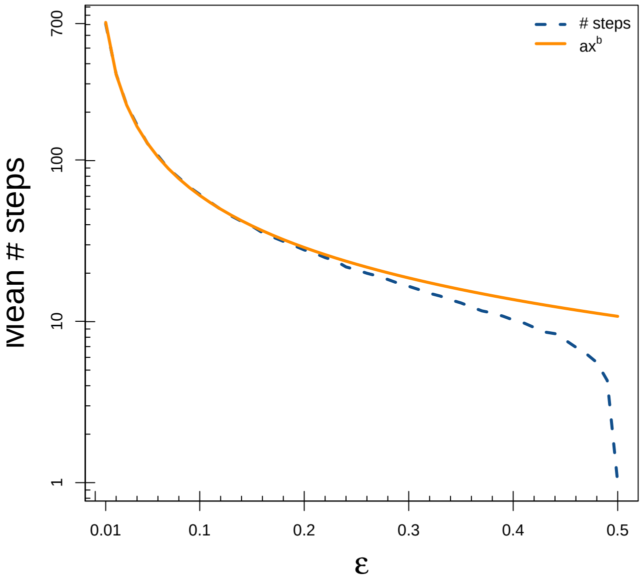

Figure S1: Estimated mean number of steps needed to get the fully concordant network as a function of ε (dashed blue curve). The results are averaged over 50 realizations. The decay of the estimated mean number of steps is best fitted by the power law ax^{-b} (solid orange curve), where the parameters $a = 5.105$ and $b = 1.072$ are obtained through (NLS) fitting.

S2 Convergence Time

Figure S3 shows the summarizing statistics for the time steps needed to reach the final state (under the different parameters combinations) by boxplots. Black horizontal lines represent the median of the number of steps needed, and the colored boxes represent the interquartile ranges (i.e., the 25th75th percentile ranges) and they statistically measure the degree of dispersion and the skewness of each analyzed distribution. Vertical lines (i.e., the whiskers) are lower and upper bounded by the minimum and maximum values of the corresponding distribution, once both outliers and extreme values are removed from the data. Individual points represent the outliers of each analyzed distribution. From the left to the right the boxplots refer ot the BCM, RBCM, and UCM.

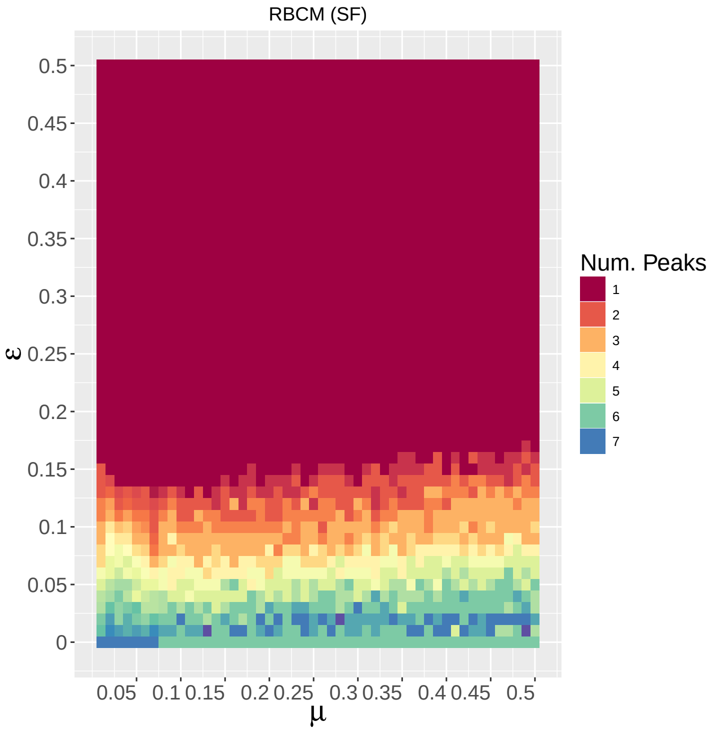

Figure S2: Final distribution of peaks for the RBCM, with varying $(\varepsilon, \mu) \in [0, 0.5] \times [0, 0.5]$. The Monte Carlo simulations are carried on a Scale-Free network with 2000 nodes for a maximum of 10^5 steps or until convergence is reached (the results are averaged over 5 repetitions).

S3 Simulation Results for ER and SW

In this section we report the simulation results for the Erdös-Rényi random network (ER) and the small-word network (SW). We perform Monte Carlo simulations of the BCM, RBCM, UCM, and RUCM on both ER and SW networks, considering $N = 2000$ nodes and the parameters $(\varepsilon, \mu) \in [0, 0.5] \times [0, 0.5]$ (the results are averaged over 5 repetitions). Given the final distributions of opinions obtained by the simulations, we compute the number of peaks of opinions as the local maxima in the distribution of frequencies of opinions. To be specific, we divide the interval $[0, 1]$ in 100 bins of length 0.01 and consider the frequencies of values falling in each interval. We regard two peaks to be separate if the distance between the middle points of the respective bins is smaller than 0.1. All the results are averaged over 5 repetitions.

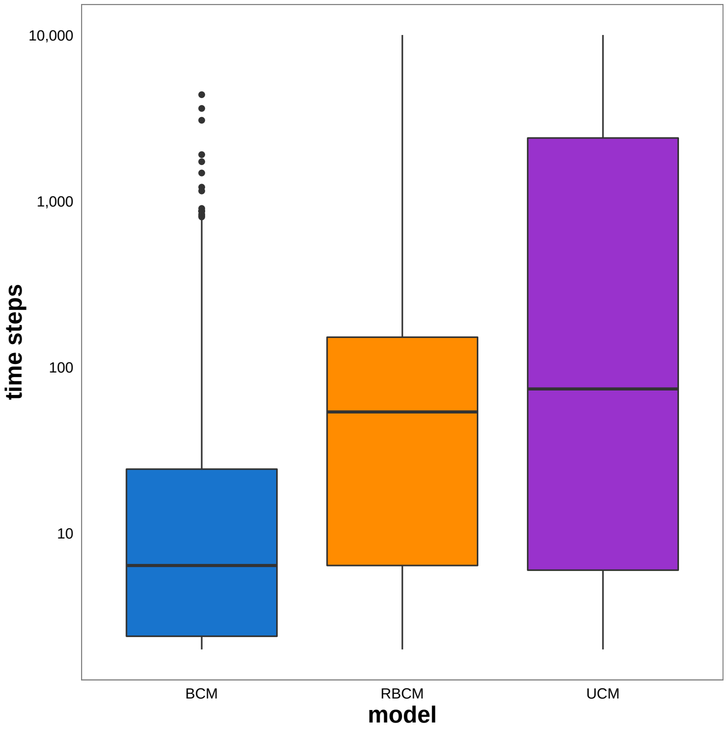

Figure S3: Boxplots of the time steps needed to reach the final state under different combinations of the parameters $(\varepsilon, \mu) \in [0, 0.5] \times [0, 0.5]$. From left to right, results for BCM, RBCM, and UCM.

Figure S4 shows the final distribution of peaks of BCM, respectively for ER (a) and SW (b), and RBCM, respectively for ER (c) and SW (d). While, Fig. S5 shows the final peaks distribution of UCM, respectively for ER (a) and SW (b), and RUCM, respectively for ER (c) and SW (d). In all cases we observe a behavior that is qualitatively similar to the SF case.

For both networks RBCM ensures a faster convergence wrt the BCM. Also, UCM and RUCM present a wide area of the parameters space in which two separate opinions coexist. For the newly introduced models we observe a similar behavior on the two different networks and on the scale-free one, in Fig. 4 of the main text.

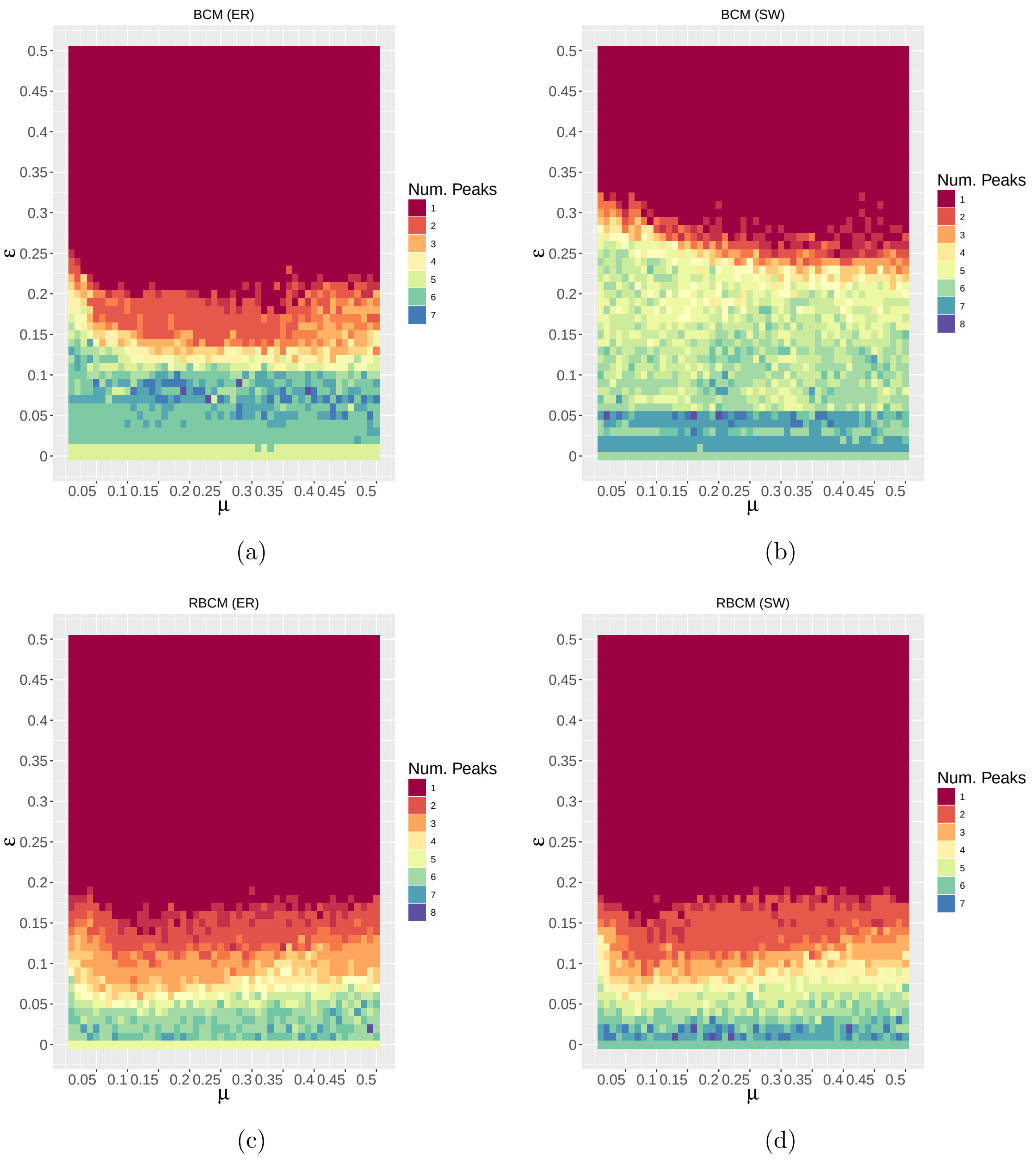

Figure S4: Final distribution of peaks for the BCM, respectively for ER (a) and SW (b), and RBCM, respectively for ER (c) and SW (d), with varying $(\varepsilon, \mu) \in [0, 0.5] \times [0, 0.5]$. The Monte Carlo simulations are carried on a Scale-Free network with 2000 nodes for a maximum of 10^5 steps (the results are averaged over 5 repetitions).

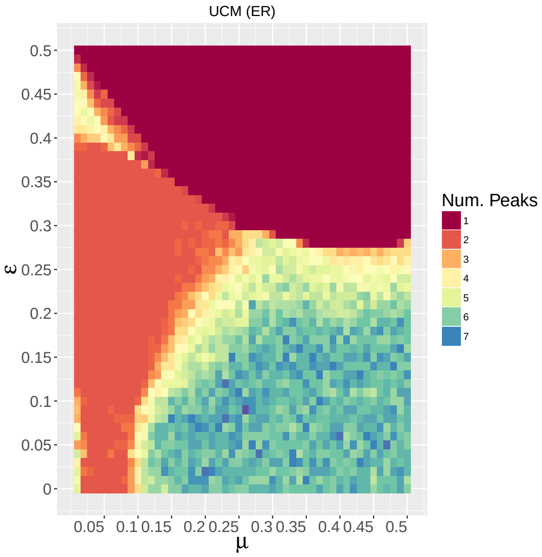

(a)

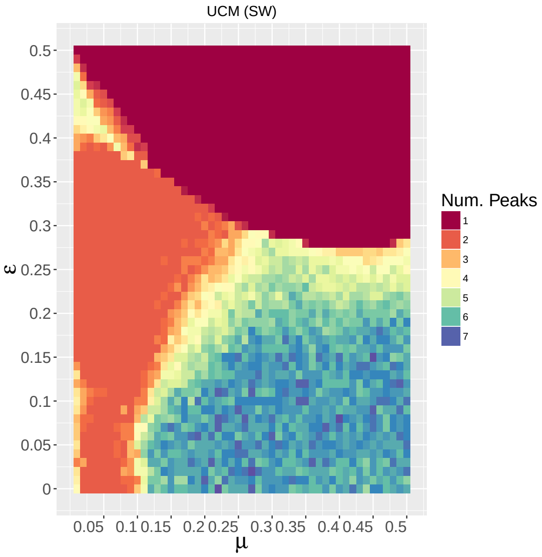

(b)

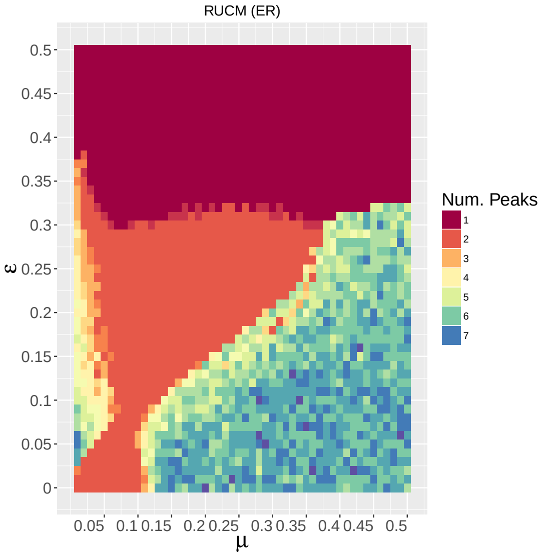

(c)

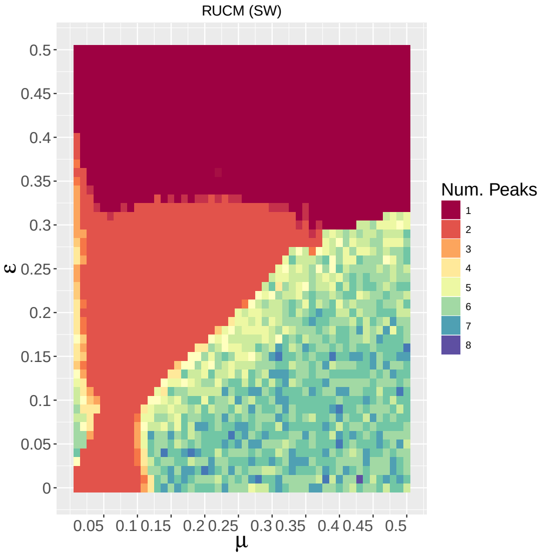

(d)

Figure S5: Final distribution of peaks for the UCM, respectively for ER (a) and SW (b), and RUCM, respectively for ER (c) and SW (d), with varying $(\varepsilon, \mu) \in [0, 0.5] \times [0, 0.5]$. The Monte Carlo simulations are carried on a Scale-Free network with 2000 nodes for a maximum of 10^5 steps (the results are averaged over 5 repetitions).
